# Preparation of Thin Films Containing Modified Hydroxyapatite Particles and Phospholipids (DPPC) for Improved Properties of Biomaterials

**DOI:** 10.3390/molecules28237843

**Published:** 2023-11-29

**Authors:** Monika Rojewska, Katarzyna Adamska, Justyna Kurnatowska, Andrzej Miklaszewski, Aneta Bartkowska, Krystyna Prochaska

**Affiliations:** 1 Institute of Chemical Technology and Engineering, Poznan University of Technology, Berdychowo 4, 60-965 Poznań, Poland; katarzyna.adamska@put.poznan.pl (K.A.); justyna.kurnatowska@student.put.poznan.pl (J.K.); 2 Institute of Material Science, Faculty of Materials Engineering and Technical Physics, Poznan University of Technology, Jana Pawła II 24, 61-138 Poznań, Poland; andrzej.miklaszewski@put.poznan.pl (A.M.); aneta.bartkowska@put.poznan.pl (A.B.)

**Keywords:** hydroxyapatite particles (HA), hydroxyapatite modification, Langmuir monolayer, surface pressure–area (π–A) isotherms, compressibility modulus, Brewster angle microscopy (BAM), XRD, FT-IR, SEM analysis

## Abstract

The main aims of thin biofilm synthesis are to either achieve a new form to promote the transport of drugs in oral delivery systems or as a coating to improve the biocompatibility of the implant’s surface. In this study, the Langmuir monolayer technique was employed to obtain films containing Mg-doped hydroxyapatite with 0.5%, 1.0%, and 1.5% Mg(II). The obtained modified HA particles were analysed via the FT-IR, XRD, DLS, and SEM methods. It was shown that the modified hydroxyapatite particles were able to form thin films at the air/water interface. BAM microscopy was employed to characterized the morphology of these films. In the next step, the mixed films were prepared using phospholipid (DPPC) molecules and modified hydroxyapatite particles (HA-Mg(II)). We expected that the presence of phospholipids (DPPC) in thin films improved the biocompatibility of the preparing films, while adding HA-Mg(II) particles will promote antibacterial properties and enhance osteogenesis processes. The films were prepared in two ways: (1) by mixing DPPC and HA-Mg (II) and spreading this solution onto the subphase, or (2) by forming DPPC films, dropping the HA-Mg (II) dispersion onto the phospholipid monolayer. Based on the obtained π–A isotherms, the surface parameters of the achieved thin films were estimated. It was observed that the HA-Mg(II) films can be stabilized with phospholipid molecules, and a more stable structure was obtained from films synthesied via method (2).

## 1. Introduction

All materials introduced into the organism, both in the form of drug carriers or as an implant, must show appropriate biocompatibility. In the course of designing drug delivery systems, we have tried to create new formulations by changing their composition and their structure. A possible and attractive solution is the development of new oral cavity drug delivery systems in the form of thin films. The efficacy of this formulation depends on the films’ composition. Oral drug delivery films have received extensive interest, as they permit faster rates of drug absorption, show higher bioavailability, and exhibit better compliance for children and elders with swallowing deficiencies [1]. Mucoadhesive and orodispersible films require proper materials as backbones that are not only capable of carrying sufficient pharmaceutical active ingredients but are also able to release these actives at a controllable rate at a desirable site, such as the buccal or sublingual mucosa. The most commonly used backbone materials are polymers or bioceramic materials, such as hydroxyapatite (HA). Currently, HA-Mg films are promising for the delivery of ciprofloxacin hydrochloride or norfloxacin [2]. Hydroxyapatite particles are a promising biomimetic alternative or supplement for oral biofilm management, as the films containing HA-Mg (II) exhibit very good bactericidal properties [1,3]. For this reason, attempts are being made to use modified hydroxyapatite HA-Mg (II) in different biomedical applications, including maxillofacial operations, dental defects, and some orthopedic applications [4,5,6]. Hydroxyapatite (HA) is the biocompatible ceramic material most often used in bone tissue engineering, as its chemical composition is similar to that of the mineral phase of bones. It has a hexagonal structure and is the most stable phase from among various calcium phosphates [7]. Synthetic HA is of interest in implantology due to its excellent biocompatibility [8,9], affinity for biopolymers [10], and high osteogenic potential [11]. Moreover, the three-dimensional structure of the HA network exhibits “flexibility” in accepting various ions to replace calcium (II) ions, including Na^+^, K^+^, Ag^+^, Sr^2+^, Cu^2+^, Zn^2+^, Mg^2+^, Bi^3+^, or Ln^3+^ [12]. Subhadip et al. [13] studied the effects of Sr^2+^ and Mg^2+^ dopants on the structural stability and biological properties of HA. They observed that the presence of Mg^2+^ and Sr^2+^ not only enhanced the stability of the particles but also improved the osteoblast response activities. Mg ion plays an important role in promoting the growth of osteoblasts and the formation of new bones, which is attributed to the fact that Mg^2+^ can activate the signaling pathway to regulate the adhesion, proliferation, and differentiation of osteoblasts [14].

The modification of implant surfaces for better osseointegration has raised increasing attention in modern orthopedic medicine [15,16,17,18]. It has been demonstrated that the osseointegration process can be influenced by a variety of factors, which may be generally divided into two categories: the environment of the bone–implant interface and the design of the implant itself [15]. The second category is focused on the properties of the material, i.e., the surface coating, topology, macrostructure, microstructure, and nanostructure of the implants. Organization of the surface coating of materials used in medicine significantly affects their interaction with a living organism, including the biocompatibility of implant materials. Wettability, the presence of functional groups, and surface roughness are the most important features determining the bioreactivity of the material surface [19]. The surface roughness on the micrometric and nanometric scale is of particular importance because it has a significant impact on the adsorption of proteins and adhesion of osteoblasts, and thus affects the rate of osseointegration [20,21]. Moreover, osteoblasts are able to distinguish and respond to differences in the chemical composition of the surface. The main inorganic component in natural bone, hydroxyapatite (HA, Ca10(PO_4_)_6_(OH)_2_), has been widely used as a bioactive coating material. Therefore, it is reasonable to expect that covering the implant surface with a film containing HA microparticles (for example) will produce a favorable response of the bone tissue.

In recent years, medication formulation into various films has become increasingly popular. The development of innovative thin films as a drug delivery platform has been pushed by several unwanted problems associated with current dosage forms, such as inconvenient administration, poorer bioavailability, and patient non-compliance [22].The most commonly used techniques for the preparation of thin films are solvent casting [23,24] and hot melt extrusion [25]; innovative techniques like inkjet printing, flexographic printing technology, or 3D printing [26] has evolved in the past few years. But the Langmuir monolayer technique enables a highly controlled formation of nanomaterial films at the molecular level [27,28,29]. The formation of Langmuir films is experimentally simple, and the technique can be easily up-scaled even for roll-to-roll production [28,30].

The Langmuir technique is universal and can be applied to any material that can float on the subphase. In particular, the Langmuir technique was preferentially employed to produce lipid or protein monolayers [28]. Currently, various biomolecules can be incorporated into the lipid monolayer, such as proteins [31], chitosan [32], collagen [33], and keratin as well as xenobiotics [34], extending the applications of Langmuir films in the biomedical field. Moreover, there is the possibility of transferring the monolayer onto a solid substrate using the Langmuir–Blodgett technique. This method allows to prepare a film consisting of several monomolecular layers on a single substrate [35,36]. The LB method offers a chance to introduce compounds with different chemical properties to the surface layer with a specific hydrophilicity/hydrophobicity, containing the appropriate functional groups that are crucial for a given type of medical material [37].

An interesting group of Langmuir films are mixed or hybrid films composed of a nanomaterial and molecules that are prepared using two main approaches. One approach is to disperse the nanomaterials in the subphase and to compress molecules at the air/water interface. Another approach is based on mixing the molecules with nanomaterials and compressing the mixture at the air/water interface. In this way, different scenarios of the behavior of nanoparticles, such as the interaction with biological systems or novel approaches to the functionalization of nanomaterials, can be investigated. Several publications have recently appeared documenting studies of nanoparticles with lipids, i.e., with silica nanoparticles [38,39], gold nanoparticles [40], etc.

The aim of our study was to obtain the Langmuir monolayers containing modified HA with 0.5%, 1.0%, or 1.5% Mg(II). Mg-doped HA was synthesized and characterized via the XRD, FT-IR, and SEM analyses.

Moreover, the films containing HA and DPPC phospholipids were studied. We expected that the presence of phospholipids would ensure a better distribution of HA particles at the air/water interface. In the Langmuir method approach, HA and DPPC molecules were spread on the top of the aqueous subphase and then compressed using moveable barriers. The obtained monolayers were characterized in detail on the basis of the obtained surface pressure (π)–area (A) isotherms and compressibility modulus (C_s_^−1^)–surface pressure (π) isotherms. The composition and structure of the monolayer were observed in situ using the Brewster angle microscopy (BAM) technique. 

## 2. Results and Discussion

### 2.1. Synthesis of HA

The wet chemical precipitation method was used to synthesize pure, undoped hydroxyapatite (HA) and Mg-doped HA powders [41,42,43]. 

Calcium nitrate tetrahydrate (Ca(NO_3_)_2_) (Sigma, Kawasaki, Japan), diammonium hydrogen phosphate ((NH_4_)_2_HPO_4_) (Sigma), and magnesium nitrate (Mg(NO_3_)_2_) (Sigma) were used as the sources of P, Ca, and Mg ions, respectively. All the chemicals were of analytical grade (>99%).

For the synthesis of HA, a 250 mL volume of (NH_4_)_2_HPO_4_ solution was added dropwise at a v = 3 mL/min and continuously stirred in a magnetic stirrer with 250 mL of Ca(NO_3_)_2_. The concentrations of the solutions were selected so that the Ca/P molar ratio was set to the stoichiometric value of 1.67. The reaction was kept in the stirrer and heated at 25 °C, and the pH at 9 was kept by adding the ammonia solution and monitored using a Lab 850 (Schott Instruments) pH meter. The obtained suspension was aged for 24 h at room temperature. In the next step, the precipitated HA was washed several times with bi-distilled water and centrifuged. The resulting powder was dried at 100 °C for 24 h and then calcined at 800 °C for 2 h (the furnace heating was 5 °C/min).

Mg-doped HA (HA-Mg(II)) was prepared via drop-wise additions of an aqueous diammonium hydrogen phosphate solution into a basic solution composed of magnesium chloride hexahydrate and calcium nitrate solution. The following steps and conditions of the synthesis were the same as for undoped HA. All final concentrations of the reagents were chosen to provide a stoichiometric (Ca + Mg)/P molar ratio of 1.67 [41].

The calcinated samples were labeled as (HA) for non-doped HA, reference material, and HA-Mg0.5, HA-Mg1.0, and HA-Mg1.5 for the samples with different Mg (II) concentrations. The reaction for the formation of synthetic HA is:

(1) $$10\mathrm{Ca}{\left({\mathrm{NO}}_{3}\right)}_{2}\cdot 4H_{2}O+6{\left({\mathrm{NH}}_{4}\right)}_{2}{\mathrm{HPO}}_{4}+8{\mathrm{NH}}_{4}\mathrm{OH}\;\to {\;\mathrm{Ca}}_{10}{\left({\mathrm{PO}}_{4}\right)}_{6}{\left(\mathrm{OH}\right)}_{2}+20{\mathrm{NH}}_{4}{\mathrm{NO}}_{3}+20H_{2}O$$

The formation reaction of the HA-Mg (II) can be expressed as [41]:

(2) $$(10\;-\;x)\;{\mathrm{Ca}}^{2+}+10\left(1\;-x\right){\mathrm{Mg}}^{2+}+6{\mathrm{PO}}_{4}^{3-}+2{\mathrm{OH}}^{-}\to {\mathrm{Ca}}_{10-x}{\mathrm{Mg}}_{x}{\left({\mathrm{PO}}_{4}\right)}_{6}{\left(\mathrm{OH}\right)}_{2}$$

The amount of Mg ions in the HA lattice was x = 0.5, 1.0, and 1.5. The detailed amounts of the reagents introduced are listed in Table 1.

### 2.2. HA Characterization

#### 2.2.1. XRD Analysis

As shown in the diffractograms, the doping of HA using Mg ions influences the hydroxyapatite structure through its incorporation into the lattice. For a small amount of Mg (II) addition, we may observe a swelling effect on the basic Ca/P hexagonal lattice, visible as a main reflex movement to the lower diffraction angle, along with the appearance of the calcium magnesium hydrogen phosphate phase. 

**Figure 1:**
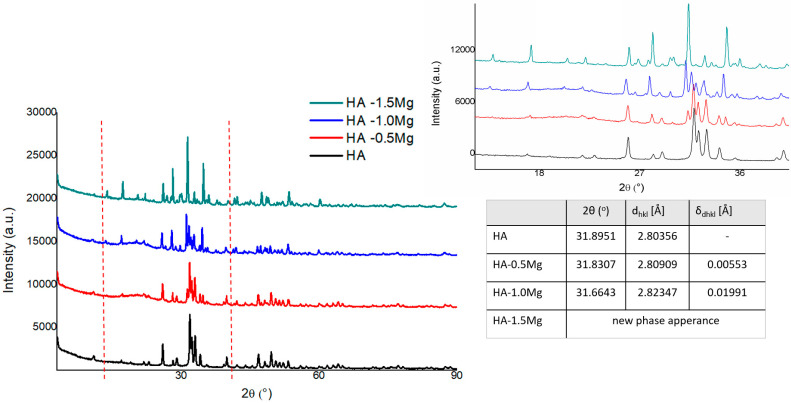
XRD spectra of HA and HA−Mg(II).

The analysis of the XRD spectra of the samples with and without Mg(II) doping indicated that Mg ions are incorporated into the elementary cells of HA (Figure 1). XRD diffractograms of the samples doped with Mg at the concentrations of 0.5% and 1.0% reveal shifts of the reflections assigned to the HA phase to lower angles, which, according to the Bragg relation, corresponds to changes in the interplanar distances in the HA structure. Figure 1 presents the XRD most intense HA single reflex analysis in obtained samples, with its varying diffraction angle position and its equivalent interplanar distance value in accordance with Bragg’s law.

Additional effects accompanying the HA structure transformation with increasing content of Mg are the appearance of the calcium magnesium hydrogen phosphate phase and the echo assigned to the amorphous structure with a maximum of 20.34 degrees. The results do not exclude the effect of the amorphous phase appearance on the lattice parameters of the base HA structure; however, more probably, the changes in the HA structure are a consequence of a complex process of structural strain effects (indirectly observed in the range of Williamson–Hall dependence) and incorporation of Mg ions in the HA matrix. With a further increase in the Mg ion content to 1.5%, the above observed trend was confirmed as the structure was stabilized as a result of transformation, leading to the formation of new phases with Mg, such as calcium magnesium hydrogen phosphate, calcium magnesium phosphate, magnesium phosphate, and calcium hydroxide phosphate, in the sequences of their formation/detection. It should be emphasized that for the sample with Mg 1.5%, no echo assigned to the amorphous phase was detected, and the diffraction reflections showed a more extended range of FWHM.

#### 2.2.2. FT-IR Analysis

**Figure 2:**
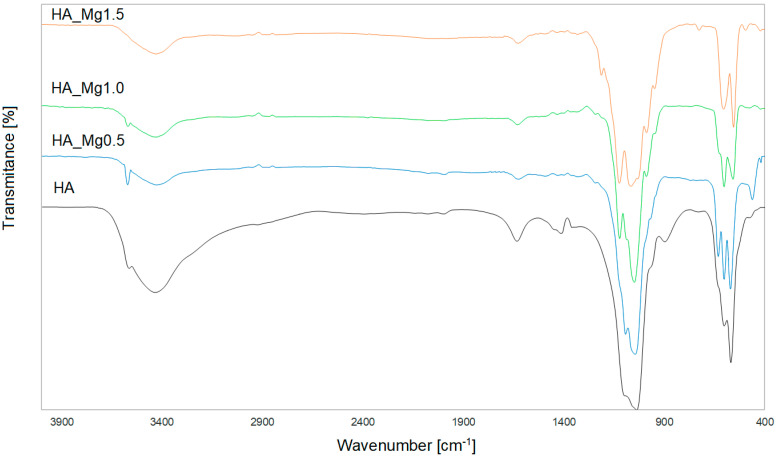
The FT−IR spectra of the HA powder and HA doped with different Mg(II) concentrations.

Figure 2 shows the spectrum of pure, undoped HA. All bands corresponding to phosphate groups visible in the spectrum were typical of the tetrahedral apatite structure [44,45]. 

The small peak that appeared at 3564 cm^−1^ originated from the stretching vibrations of the OH^−^ ions in the molecular structure of HA. The broad band at 3433 cm^−1^ and the small band at 1633 cm^−1^ were due to the bending mode of adsorbed water. The characteristic bands that correspond to the orthophosphate (PO_4_^3−^) ions, observed in the wavenumber range of 1037–1096 cm^−1^, originated from the stretching vibrations ν_3_, while the sharp peaks in the range of 568–602 cm^−1^ were associated with the bending vibrations ν_4_. In addition, the small single bands observed at 477 cm^−1^ were assigned to the bending vibrations ν_2_. The additional bands visible in the HA spectrum at 1415 cm^−1^ and 897 cm^−1^ were assigned as CO_3_^2−^ ions, which correspond to type B-type carbonation, where CO_3_^2−^ substitutes the PO_4_^3−^ groups [46,47].

The FT-IR spectra of different HA-Mg(II) powders (Figure 2) confirm the presence of the characteristic peaks of the hydroxyapatite structure, which confirms the expected results of syntheses with the addition of Mg(II) at different concentrations. 

The spectra of all materials exhibited the bands originating from the stretching and bending modes of PO_4_^3−^ groups in the ranges of 990–1100 cm^−1^ and 470–727 cm^−1^, respectively. It can be observed that for the highest concentration of Mg(II), the band assigned to the stretching PO_4_^3−^ mode was broadened (~980–1120 cm^−1^). The increasing content of Mg(II) caused a slight decrease in the intensity of the band originating from the stretching of the (OH^−^) ions, observed at around 3570 cm^−1^. This effect may have resulted from a decrease in the crystallinity of the sample with increasing content of Mg(II) [48]. The presence of the water molecule was confirmed by the broad bands at around 3426 cm^−1^ and 1630, assigned to the stretching mode of lattice water. The increasing content of magnesium ions resulted in the broadening of the peak in the range of 900–1250 cm^−1^, along with the appearance of additional small bands in this range [49].

#### 2.2.3. SEM Analysis

**Figure 3:**
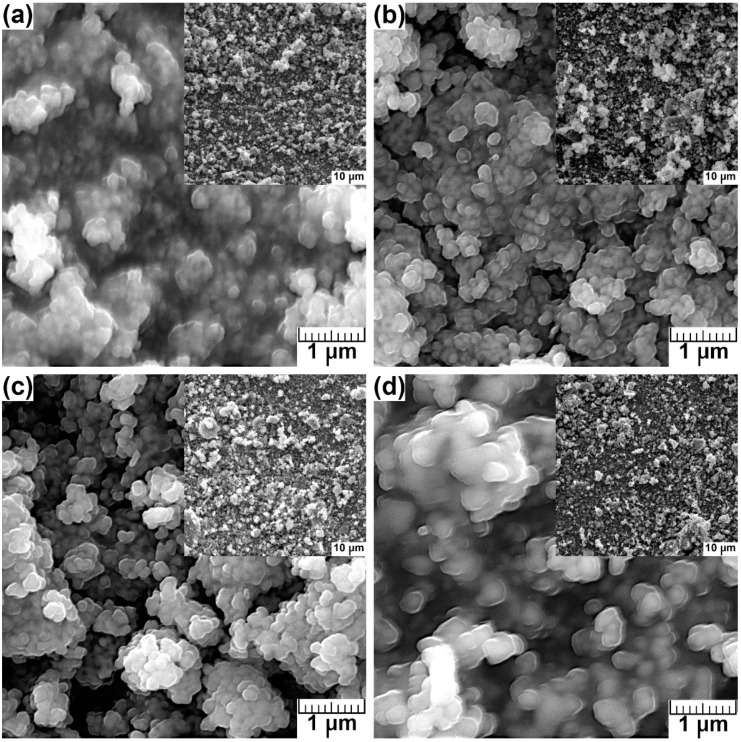
The SEM images of undoped (**a**) and HA-Mg(II) with different Mg ion concentration: (**b**) 0.5%, (**c**) 1.0%, and (**d**) 1.5%.

SEM micrographs (Figure 3) reveal the morphology and size of the synthesized powders. As shown in the SEM images, the morphology of all synthesized materials was similar. The crystallites of HA (Figure 3a) and HA-Mg(II) (Figure 3b–d) were mainly 100–200-nm-sized spherical particles exhibiting a tendency to form micron-sized clusters as well as larger agglomerates. The increasing content of Mg(II) did not change the shape and size of the particles.

The elemental analyses of the samples, performed using the SEM and EDS methods, revealed the presence of Ca, P, and Mg ions in the HA structure. EDS analyses were performed by collecting data from ten sites for each sample. The contents of Mg(II) ions were calculated as the average of the results obtained from the ten sites. The content of Mg(II) in the synthesized samples was determined to be compared with that implied via stoichiometric calculations. 

The obtained results (Table 2) indicate that the content of Mg(II) in the samples is very close to its stoichiometric amounts, except for the sample containing 1.5% Mg(II), for which it is much higher. This may be a result of the presence of other solid phases in the HA crystal lattice or the adsorption of the modifying ion (Mg(II)) on the surface. Additionally, the higher content may be due to the limited ability to replace Ca^2+^ with Mg^2+^ ions, resulting from differences in the size of the ions of these elements, as the ionic radius of Mg^2+^ is 75% of that of Ca^2+^ [50]. The ratio of (Ca + Mg) to P greater than the theoretical value of 1.67 may be due to the presence of other solid phases in the HA crystal lattice due to the incorporation of Mg(II).

### 2.3. The π–A Isotherm

The HA dispersion was dropped onto the interface and the obtained film was subjected to compression, during which the π–A isotherms were recorded. We wanted to point out that we observed that forming a film with HA-Mg0.5 is possible with a smaller volume of dispersion, i.e., 320 μL, than it is required to form a film containing HA-Mg1.0 or HA-Mg1.5 particles. 

**Figure 4:**
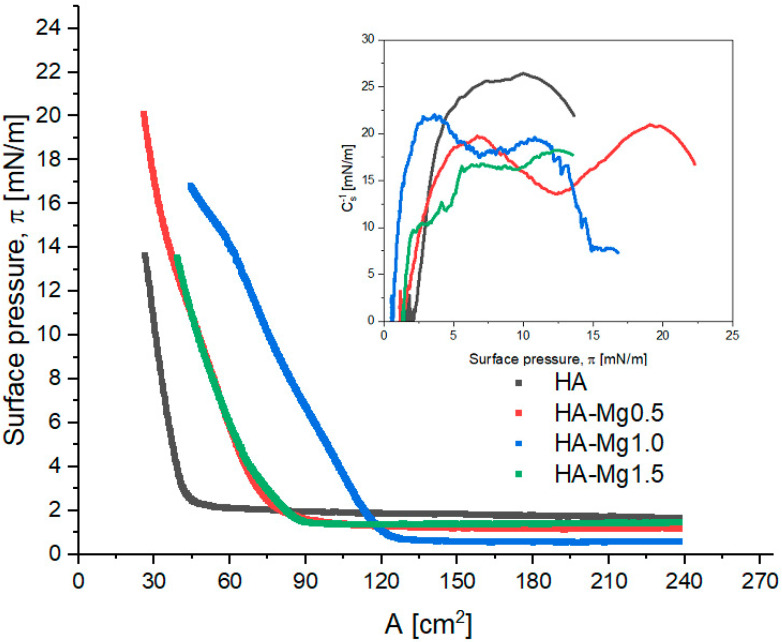
π–A isotherms of Langmuir film for HA and HA-Mg (II). The inset graph presents the surface compressional modulus (C_s_^−1^) vs. the surface pressure (π).

The results obtained for the monolayers of HA and its modification with doped Mg(II) are shown in Figure 4. The presented systems refer to an initial 400 µL of spread HA-Mg(II) solution at the interface. 

The characteristic Langmuir monolayer parameters were determined from the course of π–A isotherms and are summarized in Table 3. The collapse pressure of the studied films could not be determined; therefore, only the highest surface pressure values (π_max_) reached were shown.

The analysis of the initial surface pressure values (π_0_) revealed that the investigated dispersions exhibit different surface activities. The unmodified HA dispersion showed the highest surface activity due to its highest surface pressure at the beginning of film compression (π_0_ = 1.6 mN/m). HA-Mg(II) dispersions showed a lower level of surface activity, although no simple correlation was found between the concentration of Mg(II) and the surface activity. The presence of Mg(II) permitted the compression of the HA film to higher surface pressure values relative to those of the unmodified HA. Moreover, it was observed that the systems containing low amounts of Mg(II) formed films characterized by higher π_max_ values. 

The π–A isotherms recorded for the HA-Mg(II) films were shifted towards higher values of surface area (A). For these films, the obtained A_lift-off_ values were in the range of 75–125 cm^2^, while for the film containing only HA, the corresponding value was ca. 45 cm^2^. The presence of Mg(II) in the chemical structure of HA microparticles leads to the formation of more expanded films. The film containing 0.5% Mg(II) exhibited the greatest stability, as this structure was able to be compressed to an A_max_ ca. 26 cm^2^ at 20 mN/m. The packing degree of particles in a film is reflected in the maximum value of the compression modulus and in the character of the Cs^−1^– π curves (Table 3 and Figure 4). Moreover, the compression modulus, C_s_^−1^, provides information regarding the elasticity of the monolayers and indirectly reveals information about their physical state [51]. The compression modulus is defined as follows [52]:

(3) Cs−1=−A·dπdAT

According to the criteria outlined by Davies and Rideal [51], the state of a monolayer is classified as liquid expanded (LE, isotropic liquid) when 12.5 < C_s_^−1^ < 50 mN/m; the liquid state exists within the range of 50 < C_s_^−1^ < 100 mN/m, the liquid-condensed (LC, liquid crystalline) state exists for 100 < C_s_^−1^ < 250 mN/m, and the monolayer is described as a solid (S, 2D crystalline solid) for C_s_^−1^ > 250 mN/m.

The highest value of C_s_^−1^ (26.4 mN/m) was observed for the film containing undoped HA microparticles at 10.0 mN/m, which means that this system formed the most densely packed layer. On the other hand, the most expanded film was formed by HA-Mg1.5 microparticles, for which the max. C_s_^−1^ was ca. 18 mN/m, the trough area (A) ca. 39 cm^2^, and π ca. 12.5 mN/m. The presence of Mg(II) in the hydroxyapatite film caused a partial layer expansion, which was reflected in a decrease in the max. C_s_^−1^ value. The doping with 0.5%, 1.0%, and 1.5% of Mg(II) caused the reduction in the max. C_s_^−1^ value by 21%, 16%, and 31%, respectively. The compression modulus of the investigated systems oscillated in the range of 18–26 mN/m. Based on these max. Cs^−1^ values, it can be claimed that all the films assessed were in a liquid-expanded (LE) state, in line with the Davies and Riedel criterion [51].

**Figure 5:**
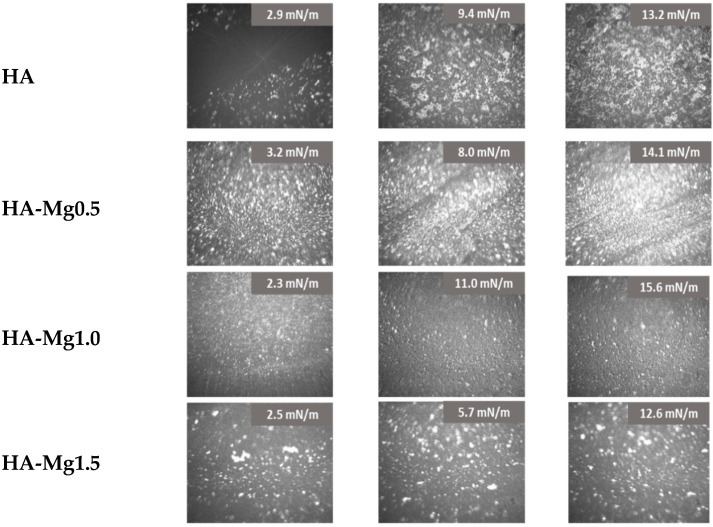
BAM images of HA-Mg(II) films.

The investigated films were visualized using a BAM microscope during the compression process. The obtained results are presented in Figure 5. HA particles were visible as white dots floating on the subphase, forming aggregates of various sizes and shapes. At a low surface pressure, the formed aggregates were located at large distances from each other. On compression, the distance between the HA particles was reduced, which resulted in an increase in the interactions between them and the formation of a more compacted structure. The formation of a highly packed film was visible for the surface pressure of 13.2 mN/m. However, this film was characterized by an uneven distribution of HA particles on the interphase.

Doping the HA systems with Mg(II) exhibited a clear impact on the film morphology, as the Ha-Mg(II) films showed a more dispersed and fine-grained structure. For the film of HA-Mg0.5, the highest number of particles at the interface was noted. Compressing this film to 14 mN/m caused the formation of a densely packed film with a visible uplift of HA-Mg particles above the subphase. On the other hand, a three-times greater amount of Mg(II) ions caused the formation of a more heterogeneous film containing numerous aggregates. The film of HA-Mg1.0 displayed a completely different morphology than those of the earlier described systems, and during its compression no large aggregates were formed. However, for the HA-Mg1.0 film, a significant shift of the π–A isotherm towards a larger through surface (A) was observed, which definitely proved that microparticles were located at the interface and formed the film.

**Figure 6:**
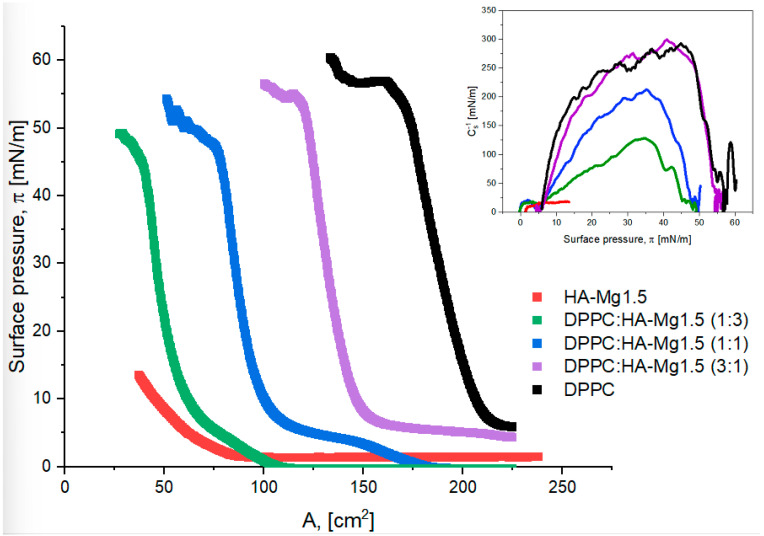
π–A isotherms of Langmuir film for HA-Mg1.5 and DPPC with different volume ratios. The inset graph presents the surface compressional modulus (C_s_^−1^) vs. the surface pressure (π).

In the next step of our study, we decided to prepare mixed films containing doped HA and phospholipid (DPPC) molecules to obtain more densely packed and stable layers. We produced mixed films containing HA-Mg1.5 and DPPC molecules at different volume ratios. Figure 6 shows the π–A isotherms recorded for the described systems.

The surface parameters characterizing the tested systems are listed in Table 4. The phospholipid film was characterized with the highest collapse pressure (π = 56.8 mN/m), which corresponded to a surface of ca. 225 cm^2^, while the HA film reached a collapse pressure of ca. 13.5 mN/m and an area of ca. 39 cm^2^. The π–A isotherm was shifted towards higher surface values with increasing content of DPPC molecules in the mixed layer.

It was observed that with increasing content of HA particles in a mixed film, the π_collapse_ value decreases. A three-fold volume excess of HA relative to that of DPPC molecules reduced the value of π_collapse_ from 56.8 mN/m to 49.2 mN/m. On the other hand, an excess of HA particles allowed for a stronger compression of the mixed film at a specific collapse pressure. For the DPPC:HA-Mg1.5 (1:3) system, the collapse surface area was ca. 29 cm^2^, while for a DPPC monolayer the corresponding value was ca. 161 cm^2^. Perhaps, HA particles contained in the mixed film diffuse into the subphase, creating voids between the phospholipid molecules. As a consequence, the mixed film can undergo extremely strong compression. It should also be emphasized that the run of the π–A isotherms obtained for the mixed films was similar to that of the π–A isotherm obtained for the DPPC film (Figure 6). Therefore, it can be concluded that the presence of DPPC molecules mainly determined the surface properties of mixed monolayers. Table 4 shows the compression modulus values for the considered systems. The highest value of the C_s_^−1^ of ca. 299 mN/m was determined for the DPPC:HA-Mg1.5 system, with a volume ratio of the components of 3:1. The structure of this film corresponded to the monolayer formation in the solid phase (S). Thus, the addition of HA-Mg1.5 particles to the DPPC film resulted in the formation of a more densely packed film relative to the packing of the monolayer consisting of phospholipid molecules. However, a higher proportion of HA-Mg1.5 particles in the mixture led to the formation of less condensed films. The films formed via the systems of 1:1 and 1:3 of DPPC:HA-Mg1.5 were definitely more expanded than the structure of HA-Mg1.5. The estimated C_s_^−1^ values were 212 mN/m and 128 mN/m for the volume ratios of the components of 1:1 and 1:3, respectively; these films corresponded to the LC state. To sum up, the formation of mixed films with phospholipids permits the retrieval of much more orderly and condensed structures.

**Figure 7:**
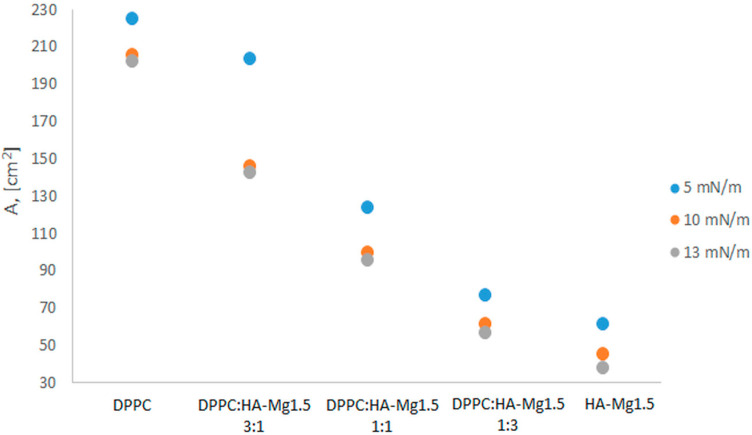
Impact of surface pressure (π) and the composition of mixed systems on the area occupied by molecules at the interface (A).

Based on Figure 7, it can be concluded that the greater the level of participation of phospholipid molecules, the higher the area occupied by molecules at the interface (A) at a given surface pressure (π). As expected, a lower surface pressure favored the formation of a looser film structure (with a greater area per molecule). At the same surface pressure, the area occupied by the molecules was larger for the mixed system with the phospholipids (DPPC) volume, being three times greater than the HA-Mg(II) dispersion’s volume. This trend was maintained for all studied films compressed at a fixed surface pressure of 5, 10, or 13 mN/m.

**Figure 8:**
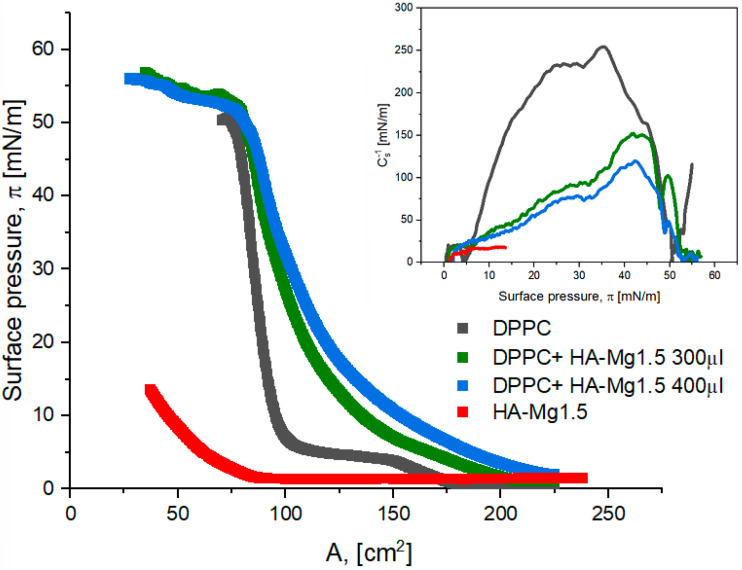
π–A isotherms of Langmuir film for HA-Mg1.5 and DPPC at different volumes. The inset graph presents the surface compression modulus (C_s_^−1^) vs. the surface pressure (π).

Another way that was employed to obtain more stable mixed films of DPPC:HA-Mg1.5 was to form a DPPC film and then deposit a dispersion of modified HA-Mg1.5 particles on its surface. We assumed that the lipid film formed was supposed to constitute a natural barrier against the diffusion of HA-Mg1.5 microparticles into the subphase and, simultaneously, enhance their accumulation in the mixed film. Figure 8 presents the π–A isotherms obtained for the analyzed systems at the dispersion volume of 300 µL and 400 µL.

The estimated values of the characteristic surface parameters are presented in Table 5. The course of the π–A curves obtained for the mixed films was similar to that of the DPPC isotherm. However, the characteristic plateau at the surface pressure of 5–7 mN/m, which corresponds to the LE/LC state of the DPPC monolayer, was found to disappear. The plateau disappeared more effectively for the mixed systems with a greater content of HA-Mg1.5. The π–A isotherms obtained for the two-component systems were shifted towards higher surface area values. A_lift-off_ was ca. 225–230 cm^2^ for the mixed films, while for DPPC the corresponding value was ca. 174 cm^2^, and for the HA-Mg1.5 film it was of ca. 86 cm^2^. The shift effect in the π–A isotherm was more pronounced for greater volumes of HA-Mg1.5 in the system. This effect proved the incorporation process of the modified HA microparticles into the DPPC film.

The π_collapse_ values obtained for the mixed monolayers (DPPC + HA-Mg1.5) were much higher than that of the film only containing HA-Mg1.5. The mixed films were more stable than the layers formed using only DPPC or dispersion containing modified HA-Mg1.5 (Table 5). Through the deposition of the HA-Mg1.5 dispersion onto the lipid monolayer, we obtained a film of the structure that corresponded to a lower Cs^−1^ value than that of the DPPC monolayer. The presence of HA-Mg1.5 resulted in the formation of an expanded phospholipid film and, finally, to the structure that corresponded to the LC state. As shown in Table 5, the used volume of HA dispersion affects the state of the monolayer formed. Deposition of a larger volume of HA-Mg1.5 dispersion led to the formation of a less condensed film.

**Figure 9:**
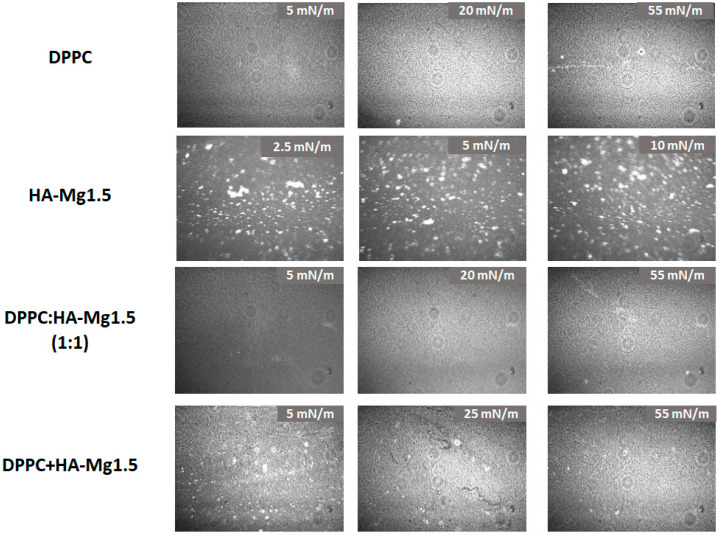
BAM imagines of the films spread at the air/water interface.

The morphology of the films spread at the air/water interface are shown in Figure 9.

The images captured during the compression clearly demonstrated the changes in films’ morphology with increasing surface pressure. The particles’ density increased, confirming the formation of a mixed film containing DPPC and HA-Mg1.5 molecules at the air/water interface. The obtained BAM images confirmed our assumptions that the method of spreading the HA-Mg1.5 dispersion (400 µL) on the DPPC monolayer promotes better retention of HA microparticles at the interface. Uneven dispersion of HA-Mg1.5 particles was observed in the mixed film structure. In the other case, when a mixture of DPPC and HAP-Mg1.5 was deposited on the subphase, a more uniform film was created with a small content of HA-1.5 Mg particles. 

## 3. Materials and Methods

### 3.1. Substances

The 1,2-dipalmitoyl-*sn* glycero-3-phosphocholine (DPPC) used in the experiments was purchased from Sigma-Aldrich. All the chemicals were of analytical grade (>99%). A chloroform of high-purity Uvasol (Merck, Krakow, Poland) was employed to prepare the spreading solutions. We used organically prepared HA (100–200 nm) and their modification with an average particle size of 450–600 nm.

### 3.2. Sample Characterization 

#### 3.2.1. XRD Analysis

The crystallographic structure of the undoped and Mg-doped HA bioceramic samples was analyzed using X-ray diffraction (XRD, Panalytical Empyrean, Almelo, The Netherlands) equipment, with the copper anode (CuKα—1.54 Å) at a Brag–Brentano reflection mode configuration with 45 kV and 40 mA parameters. The measurement parameters were set up for 3–90° with a 30 s. per a step size of 0.03° in all cases.

#### 3.2.2. Fourier-Transform Infrared Spectroscopy

FT-IR analysis was performed to evaluate the functional groups and chemical composition of the synthesized undoped and Mg-doped HA (Vertex70, Bruker Optics, Billerica, MA, USA). The samples were mixed with KBr, and the spectra were obtained over the 400–4000 cm^−1^ region (number of scans: 32; resolution: 4 cm^−1^). 

#### 3.2.3. Scanning Electron Microscopy/Energy-Dispersive Spectroscopy

The morphology of the samples was investigated using a MIRA-3 scanning electron microscope (TESCAN, Brno, Czech Republic). Prior to the SEM observation, all samples were covered with a thin layer of carbon using a JEOL JEE 4B vacuum evaporator. The thin carbon-sputtered layer was designed to reduce the effect of accumulation of electric charge as a result of the electron beam interaction with the sample surface. The carbon-sputtered layer had a 20 nm thickness, and after applying it, SEM tests were started. The tests used a high voltage (emitted from an electron gun) of 10 kV, whereas the working distance was 15 mm. Samples were analyzed under both SE and BSE contrasts.

The chemical composition of analyzed samples was investigated via energy-dispersive spectroscopy (EDS) using a UltimMax energy-dispersive spectrometer (Oxford Instruments, High Wycombe, UK). Aztec Energy Live Standard software V1 was utilized to determine the point analysis results.

#### 3.2.4. Dynamic Light Scattering Method (DLS)

Particle sizes of HA-Mg(II) were studied using the DLS method. For all samples doped with 0.5%, 1.0%, and 1.5% Mg, the obtained particle sizes were in the range of 450–600 nm, with a polydispersion index of 0.44.

### 3.3. Registration of π–A Isotherms

All reported experiments were carried out using a computer-controlled Langmuir balance (KSV Nima) equipped with a Langmuir–Blodgett trough (total area surface equal to 273 cm^2^) and two Teflon barriers, allowing symmetric compression of the liquid surface. During isothermal compression, the surface pressure was measured using a platinum Wilhelmy plate (instrumental accuracy: 0.01 mN/m). The main part of the measuring apparatus was a Teflon trough filled with ultrapure water as the subphase (18.2 MΩ·cm; 71.98 ± 0.01 mN/m). Before the experiment, the water surface was cleaned using a suction pump in order to obtain a subphase surface pressure below 0.2 mN/m at the maximum compression. The basic and widely used technique to characterize a Langmuir monolayer is the surface pressure (π)–area (A) isotherm, which is a plot of the surface pressure change (a measure of reduction in surface tension, i.e., two-dimensional analogue of pressure) as a function of the trough area available to molecules on the aqueous subphase surface. The π–A isotherm was obtained upon symmetrical compression caused by the movement of two barriers at a constant speed of 10 mm/min (3.75 cm^2^/min). Each experiment was repeated three times to ensure the reproducibility of the curves up to ±1 cm^2^ at a constant temperature of 21 ± 0.1 °C. 

#### 3.3.1. π–A Isotherm of Modified HA Microparticles

A dispersion of HA and HA-Mg(II) was dissolved in high-purity ethanol (99.8%; Sigma-Aldrich, Krakow, Poland). The concentration of the dispersion was 1 mg/mL. Before the π–A measurement, each dispersion sample was left in the ultrasonic cleaner for 30 min. Subsequently, the dispersion solution was applied to the air/water interface using a microsyringe, and following the evaporation of ethanol (10–15 min), the monolayer was compressed via moved barriers on the Langmuir trough. The volume of the deposited solution equaled 400 µL.

#### 3.3.2. π–A Isotherm of Films Containing HA Microparticles and DPPC

The HA-Mg1.5 and phospholipid (DPPC) samples were dissolved in high-purity ethanol. The HA particles with phospholipids (DPPC) were mixed at the volume ratios of 1:3, 1:1, and 3:1. Each sample was left in an ultrasonic cleaner for 30 min before being measured. The prepared mixture (50 µL) was spread evenly onto the interface using a microliter syringe. The molecular layer surface was formed after the solvent was evaporated (ca. 15 min). 

#### 3.3.3. π–A Isotherm of DPPC Monolayer and Deposited HA Dispersion

First, the phospholipid was dissolved in high-purity chloroform (UVsolv., Merck, Krakow, Poland) to provide a 1 mg/mL solution. The molecular layer surface was formed after the chloroform from the solution of phospholipid DPPC was evaporated (ca. 15 min) from the air/water interface. Then, an appropriate volume of the HA-Mg1.5 dispersion was dripped onto the formed lipid film (300 µL or 400 µL). The prepared systems were compressed in order to obtain π–A isotherms.

### 3.4. Brewster Angle Microscopy (BAM)

The BAM device (MicroBAM, KSV Nima, Helsinki, Finland) was employed to establish the films’ morphology. This technique allowed for visualizing and monitoring the structures that were formed during the compression of the monolayer evidencing, in particular, the structural modification related to the presence of microparticles [53,54].

## 4. Conclusions

In this study, we have shown that using the Langmuir technique it is possible to obtain films containing only unmodified HA and HA-Mg(II) particles. However, as can be inferred from the results, the used volume of HA dispersion impacted the state of the formed monolayer. A larger deposited volume of HA-Mg1.5 dispersion led to the formation of a less condensed film.

In general, it can be concluded that the mixed films containing HA-Mg and phospholipids are characterized by higher stability and higher degree of particle packing compared to the HA-alone film. Our results show that the addition of phospholipid molecules significantly improves the properties of the formed mixed films. It should be stressed that the possibility of forming highly condensed and stable layers is very important in the aspect of their future application. Moreover, in the applications of the thin films as coatings in implantology or as oral drug delivery systems, a very important feature is their biocompatibility. From this point of view, the addition of phospholipids as an ingredient ameliorating this property of the newly formed thin films was of substantial significance. Summing up, the use of phospholipids as a dispersing agent for HA-Mg molecules has been evidently confirmed with the results of the presented study.

## Figures and Tables

**Table 1:** The amount of reagents containing individual ions used in the preparation of undoped and doped HA. Molar concentrations of the elements used in the preparation of pure and doped HA.

Sample	Ca n (mmol)	P n (mmol)	Mg n (mmol)	Final Chemical Formula
HA	100	60	0	${\mathrm{Ca}}_{10}{\left({\mathrm{PO}}_{4}\right)}_{6}{\left(\mathrm{OH}\right)}_{2}$
HA-Mg0.5	95	60	5	${\mathrm{Ca}}_{9.5}{Mg}_{0.5}{\left({\mathrm{PO}}_{4}\right)}_{6}{\left(\mathrm{OH}\right)}_{2}$
HA-Mg1.0	90	60	10	${\mathrm{Ca}}_{9}{Mg}_{1}{\left({\mathrm{PO}}_{4}\right)}_{6}{\left(\mathrm{OH}\right)}_{2}$
HA-Mg1.5	85	60	15	${\mathrm{Ca}}_{8.5}{Mg}_{1.5}{\left({\mathrm{PO}}_{4}\right)}_{6}{\left(\mathrm{OH}\right)}_{2}$

**Table 2:** Amount of elements based on EDS analysis (wt %).

Sample	Ca	P	Mg _(Ex)_ *	Mg _(T)_ *	(Ca + Mg)/P
HA-Mg0.5	37.6 ± 2.5	18.8 ± 1.1	1.0 ± 0.1	1.2	2.05
HA-Mg1.0	36.4 ± 1.7	20.5 ± 1.3	2.4 ± 0.16	2.4	1.89
HA-Mg1.5	33.9 ± 0.8	22.7 ± 1.1	4.3 ± 0.6	3.6	1.68

* where Ex and T denote the experimental (EDS) and theoretical (stoichiometric) contents of Mg(II).

**Table 3:** Characteristic parameters of π–A isotherms: A_lift-off_—lift-off area of surface pressure, π_max_—maximum surface pressure, A_max_—area corresponding to the layer at maximum surface pressure, π_0_—surface pressure before the compression process, and max. C_s_^−1^—maximum value of the compression modulus.

System	$\pi_{0}$ (mN/m)	$\pi_{max}$ (mN/m)	$A_{max}$ (cm^2^)	$A_{lift-off}$ (cm^2^)	max. C_s_^−1^ (mN/m)
HA	1.6	13.6	26.6	44.7	26.4
HA-Mg0.5	1.2	20.1	26.0	75.8	21.0
HA-Mg1.0	0.6	16.8	44.5	125.4	22.0
HA-Mg1.5	1.4	13.5	39.3	85.8	18.2

**Table 4:** Characteristic parameters of π–A isotherms: A_lift-off_—lift-off area of surface pressure, π_collapse_—collapse pressure, A_collapse_—area corresponding to the layer collapse, and max. C_s_^−1^—maximum value of the compression modulus.

System (50 µL)	$\pi_{collapse}$ (mN/m)	$A_{collapse}$ (cm^2^)	A_lift off_ (cm^2^)	max. C_s_^−1^ (mN/m)
DPPC	56.8	161.7	225.0	292.9
DPPC: HA-Mg1.5 1:1	50.3	61.5	174.7	212.9
DPPC: HA-Mg1.5 1:3	49.2	29.1	225.0	128.6
DPPC: HA-Mg1.5 3:1	54.6	116.4	107.6	299.4
HA-Mg1.5 (400 µL)	$(\pi_{max}$) 13.5	(A_max_) 39.3	85.8	18.2

**Table 5:** Characteristic parameters of π–A isotherms: A_lift-off_—lift-off area of surface pressure, π_collapse_—collapse pressure, A_collapse_—area corresponding to the layer collapse, and max. C_s_^−1^—maximum value of the compression modulus.

System	$\pi_{collapse}$(mN/m)	$A_{collapse}$(cm^2^)	A_lift off_ (cm^2^)	C_s_^−1^ (mN/m)
DPPC 25 (μL)	57.3	59.0	174.3	254.6
DPPC + HA-Mg1.5 300 (μL)	56.9	34.7	225.0	152.0
DPPC + HA-Mg1.5 400 (μL)	56.0	27.4	230.0	119.5
HA-Mg1.5 400 (μL)	$(\pi_{max}$) 13.5	(A_max_) 39.3	85.8	18.2

## Data Availability

Data are contained within the article.

## Abbreviations

A—Langmuir trough area (cm^2^); π—surface pressure; A_lift-off_—lift-off area of surface pressure (cm^2^); A_max_—area corresponding to the maximum pressure (cm^2^); π_collapse_—collapse pressure (mN/m); A_collapse_—area corresponding to the monolayer collapse (cm^2^); C_s_^−1^—the compression modulus (mN/m); LE—liquid-expanded state; LC—liquid-condensed state; BAM—Brewster angle microscopy; HA—hydroxyapatite microparticles; and DPPC—phosphatidylcholine.
